# A comparison of preprint search aggregators: comprehensive identification of preprints in the information retrieval stage of evidence syntheses

**DOI:** 10.1017/rsm.2026.10101

**Published:** 2026-06-17

**Authors:** Zahra Premji, Sarah McGill, Amy Riegelman

**Affiliations:** 1 Libraries, University of Victoria, Canada; 2 University Libraries, University of Minnesota Twin Cities, USA

**Keywords:** evidence synthesis, information retrieval, preprints, systematic searching

## Abstract

This study investigated information retrieval of preprint records in the context of evidence synthesis work and compared 12 sources used to discover preprints. Identification of grey literature is often required or recommended in evidence synthesis guidance, and preprints are categorized as grey literature. The purpose of this work is to inform how and where to search for preprints to maximize coverage (through exploration of preprint server across aggregators and databases) while balancing search efficiency. Authors selected aggregators and databases hosting two or more preprint servers and then tested search functionality and extracted characteristics and features. Authors analyzed and compared the selected sources, tabulated the number of essential features, and created comparison tables reflecting database and aggregator features. The study protocol was registered in Open Science Framework registries. Preprint aggregators and databases differ in their content coverage, and their ability to design a comprehensive and reproducible search strategy. Limitations such as character or word limits for queries, limited advanced search operators, and missing export functionality affect the usability of aggregators for evidence synthesis searches. Ongoing updates to search interfaces and functionality and differing approaches to versioning make it challenging to study discovery of preprints across sources. The recommendations and scenarios in this article will assist searchers engaged in evidence synthesis to make informed decisions about where to search for preprints.

## Highlights

### What is already known?

- The inclusion of preprints in evidence synthesis is recommended by methodological guidance documents across disciplines.
- Information retrieval challenges have been highlighted in existing literature; however, preprint-related search functions in preprint aggregator sources have not been evaluated.

### What is new?

- This study conducted a comprehensive evaluation of search functions of preprint aggregators specifically for systematic searches.
- This study explored the volume of coverage of medRxiv, the key medical preprints server, across the aggregators.

### Potential impact for *RSM* readers

- By showcasing the present and absent search features of these aggregators, we hope to raise awareness among researchers and database/aggregator providers about the current suitability of these systems and the requirements of evidence synthesis searchers.
- We provide a discussion of considerations with the hope of assisting searchers to determine which aggregators are the most appropriate for their specific evidence synthesis projects.

## 1 Introduction and rationale

Although preprints have been disseminated for decades by mail and later via preprint servers, their use and accessibility have exploded since COVID-19, opening opportunities for research discovery in a variety of disciplines.^1^ ^–^ ^3^ There are varying definitions of preprints. The National Institute of Health (NIH) described preprints as a “complete and public draft of a scientific document. Preprints are typically unreviewed manuscripts written in the style of a peer-reviewed journal article. Scientists issue preprints to speed dissemination, establish priority, obtain feedback, and offset publication bias.”^4^ NISO is currently revising their Journal Article Versions documentation, and in a recent working document, they note the complexities defining preprints.^5^ They indicate that there is no “consistent definition across fields or across roles in scholarly communications” and that caveats and complications should be acknowledged but ultimately define a preprint as a “scholarly article that an author has chosen to make publicly available (for example, by uploading the article to a preprint server or repository that is recognized by a community), that generally has not been peer reviewed at the time it was posted, and that has not yet been accepted by a journal”(p. 9).^5^ Examples of complications and caveats include the fact that a preprint may never be submitted or accepted to a journal, or that, rather than pre-publication, a researcher may opt to share a preprint version post-publication in a journal.

As preprints have emerged as a source of evidence for systematic and other reviews, there has also been fragmentation in the number of preprint servers and ways to search for them. Preprints are one of many forms of grey literature. Leading evidence synthesis networks like Cochrane and Campbell mandate inclusion of grey literature in evidence synthesis publications.^6^ ^,^ ^7^ Searchers may have questions around efficiently selecting and searching from a diverse range of preprint sources. In the current version of *Cochrane Handbook for Systematic Reviews of Interventions*, Lefebvre et al. noted the challenges such as limited search functionality and exporting features as well as the inability to report reproducible search strategies according to best practices.^8^ Brietzke et al. underscored the value of including grey literature in evidence synthesis but indicated that it could generate challenges such as the inability to provide a reproducible search strategy therefore introducing an additional bias, that it may be “impossible to guarantee the reproducibility of the retrieval, because how many of the studies would or would not be found is subject to significant variation.”^9^ Lefebvre et al. recommended consulting the Preprint Repository Search Syntax Table to be used to assist with designing search strategies.^8^ ^,^ ^10^ This paper aims to further discuss how to best select preprint sources and search them.

We anticipate that this study will be of interest to expert searchers, who are already familiar with information retrieval concepts and advanced search functions commonly available in bibliographic databases, and conceptually understand the requirements of systematic searching (i.e., comprehensive searches for evidence synthesis reviews). Novice searchers can refer to the searching chapters and guidance documents of the various evidence synthesis organizations for an overview of these concepts.^8^ ^,^ ^11^ ^,^ ^12^ In this study, we assume that searchers will be conducting a core or “main” search of traditional bibliographic databases and grey literature as outlined in this guidance, in addition to preprint searching.

We define preprint aggregators as any searchable database or collection that combines preprints from multiple (2 or more) preprint servers, thus enabling preprints from different sources to be searched together. For selecting an aggregated preprint source, a searcher has the option of a handful of traditional bibliographic databases or database platforms, such as Medline, Embase, Preprint Citation Index on the Web of Science platform, and Scopus; and also free preprint aggregators such as Europe PMC, OSF Preprints, Lens.org, and OpenAlex, each with variable search functionality and features. This situation lands the searcher with questions such as:

- What sources exist for searching across preprint servers, and which preprint servers are covered in each?
- When searching a preprint source, what search features are available? Would the search strategy used for searching the traditional bibliographic databases have to be simplified or modified, or could the original terms and syntax be translated for use?
- If additional preprint sources are added to the traditional database search, are there features such as advanced command line searching, filtering, or exporting that would help or hinder the searcher’s usual workflow?

Previous works have developed frameworks for evaluating search systems relevant to systematic reviews and other evidence syntheses. In 2014, Bethel and Rogers developed a checklist of criteria with which search platforms could be evaluated for their ability to conduct complex searches, and used it to score three common bibliographic database hosting platforms (OvidSP, EBSCOhost, and ProQuest).^13^ A total of 55 criteria were included in the checklist, each being categorized as essential or desirable, and graded with a score ranging from 1 (insufficient) to 3 (performs well). In 2020, Gusenbauer and Haddaway conducted detailed comparisons of 28 academic and grey literature search systems based on 27 criteria, categorized as either necessary or desirable.^14^ There is some overlap between criteria and designations between these two studies, but not complete agreement.

## 2 Objectives and research question

The objective of this study is to provide a comparison of preprint search aggregators to help searchers select sources and facilitate efficient search design. We will compare search functionality in detail and provide recommendations for selecting the best preprint aggregators for efficient and comprehensive searches for potential use cases.

## 3 Methods

### 3.1 Methods overview

The protocol^15^ was informed by the authors’ extensive experience with information retrieval for systematic and other evidence reviews, including searching for preprints and reviewing preprint aggregators.^16^ ^–^ ^19^ Exploratory searches were conducted to identify other articles and product reviews related to preprint searching.

### 3.2 Selection of preprint aggregators

Preprint aggregators were selected from the authors’ past and present work evaluating preprint sources and from exploratory web searches. The selected preprint sources are listed in Table 1.

**Table 1:** Preprint aggregator sources compared Table 1 long description.

Database name		Number of preprint servers included	Free or subscription	URL if free access
medRxiv and bioRxiv combined		2 servers: medRxiv, bioRxiv.	Free	https://www.medrxiv.org/search
Embase (Ovid and Embase.com)		3 servers: medRxiv, bioRxiv, SSRN.	Subscription	
Europe PMC		34 servers: A complete list is available: https://europepmc.org/Preprints	Free	https://europepmc.org
Google Scholar		Many servers including bioRxiv, medRxiv. Total list not provided.	Free	https://scholar.google.com/
OSF Preprints		29 servers. A complete list is available: https://osf.io/preprints	Free	https://osf.io/preprints/
PubMed and MEDLINE	PubMed	Preprints must have NIH affiliation or support, and from one of 4 servers: bioRxiv, medRxiv, arXiv, Research Square.	Free	http://pubmed.gov
	MEDLINE (EBSCO)			Subscription	
	MEDLINE (Ovid)			Subscription	
Scopus		7 servers from 2017 onward: arXiv, ChemRxiv, bioRxiv, medRxiv, SSRN, TechRxiv, and Research Square.	Subscription	
Web of Science Preprint Citation Index		5 servers: arXiv, bioRxiv, medRxiv; chemRxiv; Preprints.org	Subscription	
Lens.org		Preprint server list not provided. The preprint sources can be found in the Journal or Publisher facet/filter.	Free access to the web platform for searching and exporting up to 1,000 records. Free account with additional features available for non-profit non-commercial use. For commercial use, subscription required.	https://www.lens.org/
OpenAlex		Preprint server list not provided. The preprint sources can be found in the source facet/filter.	Freemium: free access to all search features and monthly updates; subscription for hourly data updates and no API limits	https://openalex.org/

The eligibility criteria for preprint aggregators were as follows. The preprint source must:

- Be its own aggregator, providing a combined search of two or more preprint servers.
- Allow for a keyword search for preprint publications.
- Support searching in the English language (primary language of the authors).
- Provide a way to limit the results to preprints as a format/type or to specific preprint servers.
- Available as of the cutoff date of August 11, 2024.

Exclusion criteria:

- Sources that only cover another system that we are reviewing (i.e., cover records from PubMed and nothing further), so coverage overlap is not unique.
- Sources that include only preprints on COVID-19 or another narrow topic (e.g., artificial intelligence), due to limited usefulness.
- Any sources that are no longer updated (i.e., an archive).

### 3.3 List of search features

The authors prepared a list of key search features to compare: the list of extracted search features is presented in Table 2. Inspired by the work of both Bethel and Rogers and Gusenbauer and Haddaway in their detailed comparisons of academic search systems, we designated each criterion as either “essential” or “desired,” specifically for the context of conducting a systematic preprint search.^13^ ^,^ ^14^

**Table 2:** Essential features evaluated Table 2 long description.

ID	Category	Search function	Points	Description
1	Database feature	Total results displayed	1	The exact total number of results is displayed (e.g., for a search over 1,000 results).
2	Database feature	Bulk download/export	1	The ability to download all results, in one batch or multiple batches, rather than one by one. At least one test should be over 1,000 results.
3	Query development	Command line searches	1	The ability to write out a search directly using Boolean, if you know the syntax and field codes, rather than using text-only fields combined with drop-down menus.
4	Query development	High character or word limits	1	A character or word limit for the query box.
5	Query development	Boolean operators	1	Supports the Boolean operators AND, OR, and NOT, and they are logically consistent.
6	Query development	Nesting	1	Parentheses can be used to set the order of Boolean operations for sets of terms, e.g., allows for searches such as (A OR B) AND (C OR D).
7	Query development	Field searching	1	Provides a list of field codes for data fields that can be searched, or can search them using a drop-down list.
8a	Query development	Title field search	0.5	Search terms can be limited to the title field only.
8b	Query development	Abstract field search	0.5	Search terms can be limited to the abstract field only. Note: In the absence of a separate Abstract field, the ability to search Title OR Abstract will receive the full score.
9	Query development	Truncation (right-hand truncation)	1	The ability to truncate a search term with a symbol to retrieve variant endings and spellings (e.g., *) Confirm if there is right-hand truncation with a truncation symbol, automatic truncation (e.g., stem also retrieves stems, stemming, stemless, etc.), or no truncation permitted.
10	Query development	Exact phrase searching	1	The ability to search for the exact phrase (usually with the same word order, which is distinct from ANDing the words together).
11	Query development	Exact single term searching (limit stemming or lemmatization)	1	The ability to search for the exact term (i.e., ability to turn off lemmatization or stemming).
12	Query filters and limits	Can you limit to preprints	1	If the database includes multiple publication types, the ability to limit the search to only preprints.
13	Additional features	Reproducibility at different locations	1	Can the same search be executed by different people in different locations at the same time and obtain the same results.

Essential features were decided by consensus: the three authors evaluated each feature separately and designated it as essential or not, then met, discussed, and came to a consensus. Our essential criteria are based on practical needs for searching preprint sources, which are less likely to have the sophisticated search features of established academic search systems. A useful frame of thought is that preprint search systems are at a similar stage of evolution to search systems for conference abstracts or clinical trial registries. Bethel and Rogers have additional criteria as essential in order to develop complex, systematic searches efficiently.^13^ We do not suggest changing the Bethel and Rogers criteria for scholarly database platforms; rather we hope that preprint sources will continue to add more advanced features. We hope that the preprint comparison table will be a useful reference guide.

### 3.4 Essential feature comparison

To compare the search aggregators, each was given a point for every essential search feature that it included, except title field search and abstract field search which were combined into one feature (see Table 2). The maximum number of essential features is 13. We did not use points for the desired features. Note that the use of “points,” in this study, simply indicates the number of essential features a given aggregator provides. We do not suggest that these features carry equal weight and provide a more nuanced discussion of necessary features in the discussion section.

Table 3 shows the list of desired features evaluated.

**Table 3:** Desired features evaluated Table 3 long description.

ID	Category	Search function	Description
1	Query development	Multi-line search	The ability to have multiple search lines and combine them from a search history. (If this feature is not present, searchers have to create a large string for complex Boolean searches). This is different from an advanced search or search builder where multiple field searches can be combined but only one search set is the result.
2	Query development	Wildcards	Searching with a wildcard symbol retrieves all term variations where the wildcard stands for a single or zero character/s (an optional wildcard) or for a single character (mandatory wildcard) either inside the search term or at the end of a term. E.g., an?emia retrieves anemia, anaemia, if ? is an optional wildcard.
3	Query development	Hyphens	A hyphen within a search term is treated interchangeably with spaces, rather than needing to spell out hyphenated variations. E.g., does “socio-economic” find the same results as “socio economic,” or do you need to search for both terms? Note, the test included the use of“ .”
4	Query development	Proximity	The ability to search for two terms within a specified number of words of each other. Note: there are two types of proximity operators: those that place the operator between the two terms (or in some cases a phrase), and those that are written as a phrase with the operator placed at the end (and in those cases, do not allow phrases to be used in lieu of a single term within the proximity statement).
5	Query development	Truncation within a proximity string	The ability to combine right-hand truncation with proximity.
6	Query development	Controlled vocabulary (subject headings)	Preprints are tagged with human or automated controlled vocabulary for subject areas. Sophisticated search systems should be able to combine a search of controlled vocabulary with free text searching by Boolean operators (either on separate search lines or within the same line).
7	Query filters and limits	Limit to specific servers	The ability to limit the search to a specific preprint server or servers.
8	Query filters and limits	Can you exclude published preprints	Can you exclude preprints that HAVE been published?
9	Query filters and limits	Date limits	The ability to limit the search to a specific date or year range.
10	Additional features	Link to the final (journal) publication	A link is provided to the final publication (without clicking through to the individual preprint server).

### 3.5 Coverage testing

Because of the variability in reporting server coverage, and because of the possibility of selective coverage, we investigated the coverage of medRxiv preprints in each source. We found it useful to compare both the total number of medRxiv preprints in each source, as well as the total for a single year. We chose the year 2022 because 2022 should be far enough in time that fewer results would indicate that there is some kind of selection happening rather than a delay in data entry or transfer from the server to the aggregator. We hope this gives readers a sense of the potential coverage, gaps, and variability between each source.

### 3.6 Data collection process

The authors collected data over the period of April to November 2024, with the bulk of the search feature work conducted from April to May 2024, and the coverage tests in October 2024. Data were re-checked and updated if a source had a publicly shared update to its search functionality (e.g., OSF Preprints and OpenAlex). Data were extracted from the sources’ Help guides and from the authors’ testing and were recorded in Google Sheets. At least two authors independently extracted all information for a particular source and then compared the extraction for accuracy and completeness. The complete search testing instructions are available at https://doi.org/10.5683/SP3/VSML4B. Where possible and within a reasonable amount of time, the source organizations were contacted to clarify any missing information.

### 3.7 Deviations from the protocol

The original cutoff date for including preprint aggregators in the protocol was April 2024. The OpenAlex web interface was released in 2024 and by that summer it became clear to the team that OpenAlex had both a sufficient number of search features and also that it would be one of the larger sources for preprints. Given that we had not completed data extraction, we amended the inclusion date to August 2024 and added OpenAlex to the aggregator list.

The Essential feature “exact searching/phrase searching” was grouped together in the protocol but was divided into two essential features, “Exact phrase searching” and “Exact single term searching (limit stemming or lemmatization),” during data extraction for clarity and to better address the latter feature on its own.

The example scenarios were not planned during protocol development but were added to the study during the analysis and manuscript stage. These scenarios provided examples of two different disciplinary contexts, which might have different requirements; we provide a nuanced discussion of the decision-making for each scenario.

We did not conduct the comparison of preprint coverage across five topic areas. We originally intended to compare the number of preprints found for five topic searches (COVID-19, mental health, climate change, AI, and equity) across all preprint aggregators. After extracting the search features, it became clear to the team that it would be difficult to design search strategies that would be comparable across all preprint aggregators but also specific enough examples to be useful. Furthermore, with the broad differences in how many and which specific servers are included across the aggregators, we determined that the number of records from broad, single-concept strategies would not be as useful. There would also be inaccurate total numbers for some preprint aggregators like Google Scholar, so we opted to drop this section rather than comparing a partial list of aggregators. Our protocol included an optional overlap analysis of one or more specific searches, which was dependent on the topic searches being created. As the topic searches were discontinued, we were unable to complete this.

### 3.8 Results

#### 3.8.1 Search functions and features

In Tables 4 and 5, the results of the search feature tests are presented. Yes (with green shading) indicates that the feature is present or functional, No (with red shading) indicates that the feature is absent, Other (with yellow shading) indicates a partial presence, and NA (with grey shading) indicates that the feature is not available based on dependency on another feature which was found to be absent. The total number of essential features for each source is presented in the last column of Table 4 for ease of comparison.

**Table 4:** Results of essential feature testing in each aggregator Table 4 long description.

Source	No. of results displayed	Bulk export	Command line search	High word/ character limit	Boolean operators	Parentheses/ nesting	Field search	Title search	Abstract search	Truncation	Exact phrase search	Exact single term	Limit to preprints	Reproducibility	Number of essential features out of 13
bioRxiv and medRxiv	Yes	No	No	No	Yes	No	Yes	Yes	Yes	Yes	Yes	No	Yes	Yes	8/13
Embase (Ovid)	Yes	Yes	Yes	Yes	Yes	Yes	Yes	Yes	Yes	Yes	Yes	Yes	Yes	Yes	13/13
Europe PMC	Yes	Yes	Yes	Yes	Yes	Yes	Yes	Yes	Yes	Yes	Yes	Yes	Yes	Yes	13/13
Google Scholar	No	No	Yes	No	Yes	No	Yes	Yes	No	No	Yes	Yes	No	Yes*	6.5/13
Lens.org	Yes	Yes	Yes	Yes	Yes	Yes	Yes	Yes	Yes	Yes	Yes	Yes	Yes	Yes	13/13
MEDLINE (EBSCO)	Yes	Yes	Yes	Yes	Yes	Yes	Yes	Yes	Yes	Yes	Yes	Yes	Yes	Yes	13/13
MEDLINE (Ovid)	Yes	Yes	Yes	Yes	Yes	Yes	Yes	Yes	Yes	Yes	Yes	Yes	Yes	Yes	13/13
OpenAlex	Yes	Yes	No^	Yes	Yes	Yes	Yes	Yes	Other	Yes	Yes	Yes	Yes	Yes	12/13
OSF Preprints	Other	No	No	Yes	Yes	Yes	No	No	No	Yes	Yes	Yes	Yes	Yes	9/13
Preprint Citation Index	Yes	Yes	Yes	Yes	Yes	Yes	Yes	Yes	Yes	Yes	Yes	Yes	Yes	Yes	13/13
PubMed	Yes	Yes	Yes	Yes	Yes	Yes	Yes	Yes	Other	Yes	Yes	Yes	Yes	Yes	13/13
Scopus	Yes	No	Yes	Yes	Yes	Yes	Yes	Yes	Yes	Yes	Yes	Yes	Yes	Yes	12/13

*Note:* “Other” for the “Abstract field” test indicates that this search field is not available as a standalone but is available in combination with the title field. “Yes*” for the “No. of results displayed” column denotes that the number of results can be estimated from the provider filter menu.

* in the reproducibility column denotes that the test result indicated that it works, even though we are unsure that it actually does.

^Denotes that this feature is available in the API, but we classified it as a No because we limited our evaluation to the web interface.

**Table 5:** Results of desirable feature tests in each aggregator Table 5 long description.

Source	Multi-line searching	Wildcards	Hyphen = space	Proximity searching	Truncation with proximity	Controlled vocabulary assigned	Limit to specific servers	Exclude published preprints	Ability to limit by date	Links to published version
bioRxiv and medRxiv	No	Yes	Yes	No	NA	Yes	Yes	No	Yes	Yes
Embase (Ovid)	Yes	Yes	Yes	Yes	Yes	Yes	Yes	No	Yes	No
Europe PMC	No	No	Yes	No	NA	No	Yes	Yes	Yes	Yes
Google Scholar	No	No	Yes	No	NA	No	Yes	Other^	Yes	Yes
Lens.org	Yes	Yes	Yes	Yes	No	Yes	Yes	No	Yes	No
MEDLINE (EBSCO)	Yes	Yes	Yes	Yes	Yes	No	Yes	Other*	Yes	Yes
MEDLINE (Ovid)	Yes	Yes	Yes	Yes	Yes	No	Yes	Other*	Yes	Yes
OpenAlex	No	Yes	Yes	Yes	No	No	Yes	No	Yes	No
OSF Preprints	No	No	Yes	No	NA	Yes	Yes	No	Yes	Yes
Preprint Citation Index	Yes	Yes	Yes	Yes	Yes	No	Yes	No	Yes	Yes
PubMed	Yes	No	Yes	Yes	No	No	Yes	No	Yes	Yes
Scopus	Yes	Yes	Yes	Yes	Yes	No	Yes	No	Yes	No

Other* in the “Exclude published preprints” column indicates that the feature isn’t explicitly available as a limit or filter, but that it may be possible to obtain a somewhat similar result through other means.

Other^ in the “Exclude published preprints” column indicates that preprints are typically a secondary source when merged with journal-published versions, and thus are excluded by default when executing a search for a preprint source in Google Scholar.

#### 3.8.2 Coverage tests

The results of the total number of preprint records identified and the total and 2022 medRxiv coverage (number of records identified) are presented in Table 6.

**Table 6:** Coverage test results arranged in decreasing order based on total preprint coverage in each source Table 6 long description.

Source	Total preprint coverage (Oct 31, 2024)	Total medRxiv coverage (Oct 31, 2024)	Total medRxiv coverage as a % of the medRxiv coverage	2022 medRxiv coverage (Oct 31, 2024)	2022 medRxiv coverage as a % of the 2022 medRxiv coverage
OpenAlex	6,398,000	56,580	94.44%	9,988	92.40%
Lens.org	3,909,816	5,581	9.32%	334	3.09%
Scopus	2,462,629	56,785	94.78%	10,426	96.45%
Preprint Citation Index	2,438,003	38,152	63.68%	7,748	71.67%
Europe PMC	858,887	59,684	99.62%	10,517	97.29%
bioRxiv and medRxiv	315,102	*59910	*100%	*10810	100%
Embase (Ovid)	143,107	32,098	53.58%	10,430	96.48%
MEDLINE (Ovid)	31,539	5,638	9.41%	335	3.10%
PubMed	31,539	5,638	9.41%	335	3.10%
MEDLINE (EBSCO)	31,537	5,638	9.41%	335	3.10%
Google Scholar	Not available	~82,800	Not available	~6,260	Not available
OSF Preprints	Not available	Not available	Not available	Not available	Not available

*Note:* Gray highlight indicates that it was not possible to conduct the coverage test, a ~ and red highlight indicates that the results are approximate and not accurate, and the * and green highlight indicates the gold standard values for those columns.

#### 3.8.3 Source summaries

*Europe PMC - Features.* Europe PMC is a freely accessible database hosted by EMBL’s European Bioinformatics Institute. It contains scholarly and preprint records and provides a robust advanced searching interface suitable for systematic searching. It has all 13 essential features. It has few desirable features (only four) and lacks the proximity operator (and therefore, the ability to combine truncation with proximity), wildcards, multi-line searching, or controlled vocabulary. However, it is the only source in our list that allows for post-query filtering to either include or exclude preprints that have an associated publication.

*Europe PMC - Coverage*. It had the fifth highest total number of preprint records among our source list, with 858,887 preprint records discoverable as of October 31, 2024. According to its website, it provides records from 34 different servers, though coverage is incomplete for some servers (including arXiv and SSRN). It performed well on the medRxiv coverage tests, with 97.3% and 99.6% of 2022 and total medRxiv records being discoverable.

*Embase (Ovid) - Features*. Embase through the Ovid interface is a subscription database that offers advanced search functionality suitable for systematic searching. It has all 13 essential features. It also provides the majority of the desirable features, especially those related to query development. The two desirable features it lacks are a lack of linking to the published version of the preprint, and the ability to exclude preprints that have achieved publication.

*Embase (Ovid) - Coverage*. Embase includes only preprints from three servers, medRxiv, bioRxiv, and SSRN (which was added since our coverage tests were done). Based on the coverage tests, its coverage of medRxiv appears to be relatively complete in recent years. In our coverage tests, we were able to discover 10,430 records, which is 96.5% of the 2022 medRxiv records. But coverage of records from previous years may be incomplete, as it appeared to contain only 53.6% of the total medRxiv records. Furthermore, given that it only contained bioRxiv and medRxiv at the time of our coverage test, its coverage, if complete, should match closely to that of medRxiv/bioRxiv, but at 143,107 total preprint records, it is less than half of what is found in medRxiv/bioRxiv (315,102 records on the same date).

*MEDLINE (through Ovid, EBSCOhost, or PubMed) - Features*. MEDLINE through both the Ovid and EBSCOhost interfaces (established subscription databases) provide all of the 13 essential features. Furthermore, both also lack the same two desirable features: controlled vocabulary applied to preprint records and the ability to exclude published preprints (which we scored as “other,” and is explained further in the discussion section).

PubMed is a free-to-use interface that allows for access to MEDLINE content as well as some additional content.^20^ It also provides all 13 essential features, despite the Abstract field not being separately searchable. Instead, there is a combined Title and Abstract field which can be searched. In addition to the two features that the Ovid and EBSCOhost versions of MEDLINE lack, PubMed also lacks two additional desirable features, namely, the ability to combine truncation with its less flexible proximity operator, and a single-character wildcard.

*MEDLINE (through Ovid, EBSCOhost, or PubMed) - Coverage.* Medline, through any platform, has the same preprint coverage which is primarily content from the servers bioRxiv, medRxiv, arXiv, and Research Square, though the preprints must meet NIH’s eligibility criteria.^21^ The medRxiv coverage tests we conducted proved that total coverage was only 9.41% and 2022 coverage was 3.1% of what is available in medRxiv. This shows that coverage is not complete from any of these servers, making MEDLINE an inadequate source for searching preprints comprehensively. Since the criteria are the same for all of the servers included in MEDLINE/PubMed, we can expect similarly incomplete coverage for the three other servers (i.e., bioRxiv, arXiv, and Research Square).

*Preprint Citation Index - Features*. The Preprint Citation Index is a subscription database hosted on the Web of Science (Clarivate) platform. This source has all of the 13 essential features. If users are familiar with the Web of Science platform, it will be an easy learning curve to navigate the Preprint Citation Index search functionality and bulk export features. It lacks two desirable features, namely controlled vocabulary and the ability to exclude published preprints.

*Preprint Citation Index - Coverage.* Currently, there are five preprint servers that are discoverable in Preprint Citation Index: medRxiv, bioRxiv, chemRxiv, arXiv, and Preprints.org. Since Preprints.org does not have disciplinary restrictions, a search in Preprint Citation Index would prompt results spanning many disciplines. In our coverage test for medRxiv, only 63.68% and 71.67% of medRxiv content were discoverable when we compare total and 2022 results, respectively. We confirmed with Clarivate representatives that this discrepancy is due to FRBRizing wherein preprint records for the same work are merged and the latest versions are shown.

*Scopus - Features.* Scopus is an established bibliographic database that requires a subscription to use and has the majority of the essential features on our list. It has 12 out of the 13 essential features. It is missing only one essential feature, which is the ability to bulk export preprint records resulting from a search. Of the 10 desirable features in this study, Scopus is missing only three, specifically, controlled vocabulary, linking to a published version of a preprint, and the ability to exclude published preprints. However, this is not unusual, as these three features were absent in many of the aggregators in our study. Overall, while Scopus scores well in terms of query development features, and misses desirable features that are relatively rare, the missing bulk export feature which is essential for evidence synthesis researchers makes it an undesirable option for preprint searching.

*Scopus - Coverage.* Scopus includes records from seven preprint servers, covering the health, science, and social science disciplines, with coverage starting from 2017. When tested on October 31, 2024, Scopus had a total of 2,462,629 preprints which was the third highest of all the sources we studied. In coverage tests of medRxiv specifically, it contained 94.78% of the total medRxiv coverage, and 96.45% of the 2022 medRxiv coverage. This confirms that the medRxiv coverage is relatively complete.

*OSF Preprints - Features.* OSF Preprints is a product from the Center for Open Science. This source is lacking several essential features, providing only nine of the 13 essential features. Testing indicated that result totals under 10,000 had the exact number of results displayed, whereas results totals over 10,000 did not display an exact number on the main screen. In the facet filtering options on the left side of the page, users are able to open the limit by “provider” tab to see a more granular depiction of search results by preprint server. Users are unable to bulk export search results and cannot perform command line searching. Boolean operator functionality is not intuitive but a help page clarifies that the pipe character | functions as an OR Boolean, + serves as AND, and - serves as NOT.^22^ Searchers are unable to search specific metadata fields. For desirable features, OSF lacks multi-line, proximity, wildcard searching, as well as the ability to exclude journal-published preprints. Lacking many essential features, it is suboptimal for discovery of preprints compared to other options.

*OSF Preprints - Coverage.* OSF Preprints, as a platform, includes records from 29 preprint servers, with coverage starting from 2016. Confusingly, one of the listed preprint repositories is also called OSF Preprints. Currently, only OSF-hosted content is discoverable via their search feature, and medRxiv is not among this list; therefore,x we could not complete the medRxiv coverage test. The preprints discoverable in a search of OSF preprints represent many disciplines.

*Lens.org - Features*. Lens.org is a free and open patent and scholarly source by Cambia. It is searchable using an Application Programming Interface (API) and an open web platform, with the latter being the one that was tested in this study. Preprints in Lens can be found in the scholarly works section, using the Document Type filter. It provides all 13 essential features. The default settings, however, may not be ideal for evidence synthesis searchers. Specifically, stemming is automatically turned on but can be toggled off from the “Query Tools” menu. It also does well for the desirable features. While it does allow proximity searching, its proximity operator is not as versatile as other sources, and one cannot combine truncation with a proximity string. Other than that, the only features it is missing are the lack of direct linkage to the final published version which therefore means it is not possible to exclude published preprints. Exports up to 1,000 records are available to everyone without requiring an account. Free accounts are available for non-profit non-commercial use, which provide additional features including higher bulk download capabilities. For other users, a paid subscription is required to get account access.

*Lens.org - Coverage.* Lens includes many preprint servers, but a complete list is not available. It lists PubMed and OpenAlex as some of its sources, which can be confusing, given that OpenAlex contains a greater number of preprints than Lens. The specific repositories may be listed under the “Journal” filter, where one can find arXiv, medRxiv*(partial), bioRxiv*(partial), Research Square*(partial) listed, or the “Publisher” filter, where Research Square, Center for Open Science, Authorea, PeerJ, and others are listed. As of October 31, 2024, there were 3,909,816 preprints in Lens, making it the second largest source in our study. However, its coverage of medRxiv appears to come from PubMed, as it has approximately the same coverage amounts of total (5,581, or 9.32%) and 2022 coverage (334, or 3.09%). Given the challenges with searching for and exporting preprints from the Center for Open Science repository, Lens is a desirable option to access that specific collection, while also providing access to select other repository holdings. Note that this includes all preprint records on the OSF Preprints platform from 29 servers, only one of which is the OSF Preprints repository. There does not appear to be a separate facet that would allow a user to limit further to only a single server such as PsyArXiv or OSF Preprints (the repository).

*Google Scholar - Features.* Google Scholar is an academic search engine that allows for searching scholarly articles, theses, books, and other scholarly documents, from publishers, professional societies, repositories, and universities.^23^ Google Scholar only has 6.5 of the 13 essential features, lacking key features such as displaying the exact number of results found, bulk export options, a high enough character limit, reliable nesting and use of parentheses, abstract search, truncation, and the ability to filter specifically to preprints. It provided three desirable features – the ability to limit by publication date, to limit to specific preprint servers (with limitations, as mentioned below), and links to the journal’s published version – and lacked all other desirable features. Searches that limit to preprint sources (using the relevant search field) automatically exclude preprints that have been grouped together with a corresponding journal-published version, and there is no way to turn this feature off. It should be noted that we are aware that other studies have indicated that Boolean is not reliably supported by Google Scholar.^14^ This may indeed be true; however, using our specific test instructions, Google Scholar appeared to produce the results we would have expected from working Boolean operators. Additionally, in our single reproducibility test with searchers in different locations, Google Scholar showed consistent results, which was a surprising observation. Another study found that Google Scholar’s replicability tests were sometimes successful but failed at other times.^14^

*Google Scholar - Coverage.* There is no known way to limit to preprints specifically, except by limiting to the source (server or repository) of preprints, which can cause problems if a repository indexes preprints as well as other formats of information. It is for this reason that we were unable to estimate the total number of preprint records discoverable via Google Scholar. And, even though we still attempted to conduct coverage tests of medRxiv in Google Scholar, it is clear that the number of results displayed is not accurate (as has been previously reported in Bramer, 2016).^24^ This was evident from the results of the total medRxiv coverage test, where Google Scholar over-reported ~82,000 results, but the true value (as validated from medRxiv) was 59,910 records.

*bioRxiv/medRxiv - Features*. bioRxiv and medRxiv can be searched independently or jointly in this source. For essential features, bioRxiv/medRxiv has eight out of the 13 features on our list. While containing many of the essential features, searchers are unable to bulk export or perform command line searches. There is a 128-character limit. bioRxiv/medRxiv contains some desirable features but lacks proximity, multi-line searching, and the ability to exclude published preprints. The lacking essential features are important, and their absence hinders the ability to build a systematic search query.

*bioRxiv/medRxiv - Coverage.* Only two preprint servers are searchable in this source. Its discoverable content represents medical and health sciences and biological sciences. Coverage is complete for both bioRxiv and medRxiv as it is the content host. When there is a known peer-reviewed version, it is linked in the bioRxiv and medRxiv records.

*OpenAlex - Features.* OpenAlex is a free and open tool that contains bibliographic and preprint records. Similar to Lens, it can be searched using an API or via a web interface, the latter being what was tested in this study. OpenAlex offers a premium option which provides hourly data updates via API and no limits on API calls. The OpenAlex web interface and search functionality went through a refresh in February 2026. It provides 12 of the 13 essential features, only lacking a command line search (in the web interface). While the web interface does not have command line searching, the API does, which we note here for readers’ benefit, though we did not accept it for fulfillment of this criterion. Furthermore, OpenAlex has a 2,048-character limit for queries. We judged this as being sufficiently high and thus scored it positively for this feature. Note that exact single term searching in OpenAlex is applied to the entire search using a toggle to either allow or disable stemming. This feature cannot be selectively applied to only some terms within a search string. For desirable features, it provides the ability to limit to specific servers and publication dates, proximity searching, and wildcards, but lacks multi-line searches, controlled vocabulary, links to the final publication, and the ability to exclude published preprints.

*OpenAlex - Coverage.* OpenAlex had the highest coverage of preprints among the sources we tested. As of October 31, 2024, it contained 6,398,000 preprint records. A complete list of preprint servers was not found. Similar to Lens, one can locate a list by limiting to preprints (using the Type filter) and then looking at the list of “Sources.” The top five preprint sources listed by coverage are arXiv, HAL (Le Centre pour la Communication Scientifique Directe), RePEc : Research Papers in Economics, Research Square, and bioRxiv. In medRxiv coverage tests, OpenAlex had 94.44% and 92.4% of the total and 2022 coverage compared to the original server counts. Despite lacking a command line feature and some of the desirable features, its size, coverage, and free access make OpenAlex a viable and valuable option.

## 4 Discussion

### 4.1 Connecting our work to what came before

Despite methodological guidance documents promoting the inclusion of preprints in evidence synthesis, little research has been done on searching and identifying preprints for evidence synthesis.^8^ ^,^ ^25^ Previous works have reviewed characteristics of preprint servers; however, information retrieval functions were not covered.^26^ Other articles have talked more generally about preprints and some challenges related to searching, reproducibility, or exporting.^9^ ^,^ ^27^ The study on the search functions of academic search systems, mentioned in the introduction, included many of the subscription platforms such as Ovid, EBSCO, Web of Science, and Scopus, but not freely accessible aggregators such as Europe PMC and Lens.org.^14^ A recent article discussed the experience of selecting a preprint source for a Long COVID-19 review and some of the challenges with searching.^19^ However, no previous study has systematically examined search functions across multiple preprint aggregators as well as functions specific to searching preprint records (such as linkage to the final publication and limiting to specific preprint servers). In addition to the results presented above, we discuss below some details of challenges we encountered both within our study and for working with preprint records, in general.

### 4.2 Challenges

The primary objective of this study was to evaluate the search functions of aggregator systems that index preprint records, especially those that are not on established subscription database platforms. We developed testing instructions for each of the search functions being evaluated, and recorded the responses as “yes” or “no” based on whether the result of the test behaved in a way that matched what would be logically expected from the working function. We also noted additional details where required, such as export formats available, commands to limit to preprints in each source, etc.

Testing the coverage was a secondary objective and exploratory in nature. We had initially planned to test coverage by constructing search strings for a set of topics and report the number of results for the various topic search strings per source. This would have required identifying the minimum set of search functions that were present in all sources and constructing a systematic search using only those functions. For this reason, we completed the work related to the primary objective first. After confirming what functions were available in each of the aggregator sources, we realized that some basic query-building search functions were not available in all sources (e.g., lack of reliable functioning of nesting/parentheses in two sources, inability to turn off lemmatization in two sources, lack of a truncation operator in two sources, and so on). We therefore recognized that we would not be able to construct a search string that was comprehensive and representative of a systematic search for an evidence synthesis review while being syntactically similar enough to run identically across all sources for a fair comparison of coverage for various topics. We changed the plan and tested coverage of one server that was common to the majority of the aggregators. MedRxiv was available to some extent in all but one source (OSF Preprints), so we explored the volume of medRxiv records available in each source as a way of measuring completeness. We also captured the total number of preprint records identifiable in each source to show the total size of their preprint collections.

We did not anticipate the different ways that some aggregators would handle versioning, which impacts the volume of results retrieved and may underestimate coverage in a volume-based coverage test such as the one we conducted. A clear example of this is Web of Science Preprint Citation Index where various versions of the same preprint record are consolidated into one record. A 2024 article describing preprints in Europe PMC provides detailed information about their process which, similar to Web of Science Preprint Citation Index, links together multiple versions of a preprint and only the most recent version is displayed in the search results.^28^ The article points out that some preprint servers assign a single Digital Object Identifier (DOI) to a preprint, which does not change when an update or new version is created, and all versions are linked back to the same DOI. But other preprint servers assign a new DOI to each version. In that case, the aggregator’s process would determine whether consolidation occurs or not. Despite the article stating “when a new version is added, only this latest version will be indexed and available in search results,” our coverage tests showed that Europe PMC contained 99.6% of the total number of records identified in medRxiv (p. 10).^28^ We recognize that there are other ways to test coverage, and we look forward to future studies that evaluate coverage in greater depth, and using different methods, than we did.

Aggregator sources may be useful for searching multiple preprint servers at the same time, or may simply be desirable for their search and exporting functionality. For example, all of the preprints from the Center for Open Science (OSF Preprints) platform are available through Lens.org, and even if that were the only preprint repository of interest for a searcher, using Lens.org (which has all 13 essential features) is an obvious choice compared to the original repository’s interface (which only has 9 of the 13 essential features).

As legacy for-profit publishers have taken financial interest in other aspects of the research lifecycle (such as preprints), the search landscape is changing. For example, Research Square was launched in 2013 and acquired by Springer Nature in 2022.^29^ Scopus and Social Science Research Network (SSRN), a working papers repository, are both products of Elsevier/RELX. Clarivate owns the Web of Science platform as well as the Preprint Citation Index. We may see larger for-profit subscription services bolster their indexing and aggregation of preprints.

#### 4.2.1 Challenges building search queries

As shown in our comparison of essential features and reporting of desirable features, there is variance across preprint sources and some are difficult to use in a systematic and reproducible way. The variability across platforms can be difficult to interpret even via rigorous testing. Syntax can also change. The sources used in this present study range from providing very detailed help/user information that is updated regularly to suboptimal and opaque explanations of search functionality. We advocate for search syntax changes to be clearly communicated to users and archived publicly. As written about by Barrick and Riegelman (2021), lack of communication “reduces the transparency of the search algorithms and, by extension, may reduce the reproducibility of the study” (p. 985).^30^ In the case of evidence synthesis methodologies, search strategies need to be transparently reported to ensure reproducibility. Evidence synthesis searchers, adhering to best practices, report the date(s) when searches were conducted and “a syntax change could affect the number of search results from one day to the next. When search platforms act as a blackbox, searchers and potentially evidence-based practice and policy are at a disadvantage” (p. 987).^30^

Experienced searchers know that not all evidence synthesis searches are the same. Some topics can be searched using a simpler (but still comprehensive) search strategy employing few advanced operators. In such cases, it may be possible to spell out all possible endings of a keyword stem then construct the search strings using a combination of more basic functions such as nesting, Boolean operators, forced phrase searching, and field searching. Where the above is a possibility, an aggregator like OpenAlex, which has excellent coverage and has the largest number of preprint records, may be a suitable choice. However, some topics require more complex search strategies and, in those cases, even the full suite of essential features from our list may not be sufficient to create a sensitive search. Such complex searches may require a number of desirable features such as wildcards, proximity, or the ability to use truncation with proximity. It is up to the searcher to determine what minimum combination of features is necessary for a given project. In situations where a complex topic must be searched in an aggregator that does not offer the required essential or desirable features, the searcher may be forced to create a simpler search, which differs significantly from their other database searches. In those cases, they should provide a search narrative explaining their choices.^31^

#### 4.2.2 Challenges with filtering and exporting the records

Searches for evidence synthesis value comprehensiveness and sensitivity over specificity, and for this reason result in large volumes of search results (often in the hundreds or thousands of records). Furthermore, in order to facilitate dual screening and effective and transparent data management through the study selection process, the ability to export all records resulting from a search is a critical feature. The availability of batch exporting, or lack thereof, may influence the searcher when deciding on sources. The results of this study confirmed that both OSF Preprints and Scopus do not allow batch exporting of preprint records, thus reducing their usefulness as potential sources for evidence synthesis searches. As of July 19, 2025, the preprints results tab in Scopus is still labeled as “Beta,” so it is unclear if more improvements or features will be added and whether the ability to export the results will be available in the future. This finding was surprising because batch exporting is available for bibliographic records in Scopus. However, even sources that have batch exporting options differ in the size of the batch allowed or the file formats.

The best option would be a high download limit, and this was available from a few sources we tested, including OpenAlex (100,000 records), Europe PMC and Lens.org (50,000 records), PubMed (10,000 records), and EBSCO Medline (25,000 records using the email option in both the new and classic user interface, but this may vary by institution). In the majority of systematic searches, these limits would allow a searcher to export all search results in a single file export. Where the size of the allowable batch is too small to allow for a single export, it is helpful to be able to specify the range of records to be exported. The Web of Science platform (for Preprint Citation Index) and Ovid platform (for MEDLINE and Embase) are two examples where specifying record ranges allow complete exporting of the results in as many batches as is necessary. Where a search is larger than allowed by these limits and where there is no ability to specify the range of records to export, one may be required to break down the total results into specific publication year ranges in order to stay within the allowable export size.

BioRxiv/medRxiv is different and does not have an export limit listed; however, it does not allow specification of a range or the selection of all records in the search. It does provide an option to add all citations on a page to a list with a maximum of 75 results per page. To go to the next page, one must scroll to the bottom of the page where page navigation can be found. Then, once as many pages of citations as desired are added to the list, it can be exported in a single batch. The authors tested an export of up to 500 records successfully but experienced some time-out issues with exports above 1,000 records.

Google Scholar is also challenging when it comes to viewing and export options. It has less robust options for exporting: one must either click on individual records to add them to a list before being able to batch export them or use a scraper tool such as Publish or Perish.^32^ Even with the use of a scraper tool, the limit of 1,000 visible records remains – this is the maximum number of accessible results – and anything beyond that would require the use of limits (such as publication date) to segment the search into sets of less than 1,000. Add to this the fact that Google Scholar only estimates the number of records resulting from a search query,^24^ and it becomes extremely challenging to manage exporting records efficiently and reproducibly. While we did not examine the metadata of exports from each of these aggregators, it is commonly known that exports from Google Scholar do not contain the complete abstracts for each record.

#### 4.2.3 Coverage-related challenges

When selecting databases and aggregators, searchers will need to know the date ranges of included preprint servers and whether coverage is selective or complete. In some of the sources that we explored, this information was undisclosed. We also observed errors in metadata which complicated any attempts to understand comprehensiveness as it pertains to coverage dates. Database and aggregator ingesting frequencies also influence comprehensiveness. While we did a volume test for medRxiv, further research is needed to analyze coverage and how platforms respond to versioning.

During the conduct of our study, we noticed that the level of indexing and the quality of the metadata varied across sources. Some of these quality issues may be temporary or may only impact the newer aggregators. It is unclear how much cleaning of metadata, if any, is occurring behind the scenes at some of these aggregators. For example, we noticed that the different preprint servers were not always listed within the same facet (source, repository, journal, publisher, etc.) in some aggregators. We observed different numbers of results when searching the name of the preprint server using the journal versus publisher field tags in Europe PMC. Similarly, in Lens.org, some preprint servers, such as Research Square or Authorea, Inc. were listed under “publisher,” while others, such as arXiv, were listed under “journal.” Furthermore, arXiv was not listed as a single journal: rather, there were well over 50 different titles such as arXiv: Robotics, arXiv: Learning, and many others in addition to arXiv (Cornell University) which had the greatest number of records.

A separate issue related to preprint servers, in Lens.org is that one cannot use the filtering menu to limit a search to both Research Square and arXiv at the same time since the various filters are automatically ANDed together when applied. It is possible to use the query text editor to combine different fields (Publisher and Journal) using an OR Boolean operator (e.g., (source.publisher: “authorea”) OR (source.title: “arxiv”)). Note that in the example presented, the filter facet is called “Journal” but the field tag is called “source.title,” and this variation in terminology in different parts of the interface can pose its own challenges.

The examples provided above underscore the value of the command line search functionality, which often offers more options than what may be available via filter menus. This is one of the reasons why a command line search was considered an essential function in our study.

#### 4.2.4 Challenges with connecting preprints to published versions

Linkage to the final peer-reviewed publication was available in eight of the 12 sources including bioRxiv/medRxiv, MEDLINE (EBSCO), MEDLINE (Ovid), Europe PMC, OSF Preprints, PubMed, Google Scholar, and Preprint Citation Index. But only one source, Europe PMC, provides the functionality to filter out preprints that already have a published article. Whose responsibility it is to locate and link to the final journal published varies by source. Some servers are dedicated to the practice of checking for peer-reviewed versions (e.g., bioRxiv and medRxiv), whereas other preprint servers rely on authors to submit a peer-reviewed, published DOI. Europe PMC relies on both the information from the servers it aggregates as well as its own process which is applied to records that have a PubMed ID.^28^ We applaud Europe PMC for providing transparent and detailed information about how preprint records are ingested, processed, and managed.

Another consideration is the way that published versions are linked or noted appears differently in these aggregators:

- In Europe PMC, a notice appears on the page of an individual record that “A journal published article is available.” There is no indication of this on the aggregate search results pages, so this cannot be identified when scrolling the results of a search until one goes into a specific record.
- In Ovid MEDLINE, the link to the journal-published version is in the “Title Comment” field, and records that link to an updated version of an article can be searched using one of two field codes, either the .cm (comments) or .tc (Title comment) using the following text “Update in”.cm or “update in”.tc since this common phrase of text precedes the citation itself. It should be noted that this field simply provides a citation to any linked updated article, not only published versions of preprints.^33^ However, for preprint records in Ovid MEDLINE, the majority of the linked articles should be for the published version of the article. Customizing the display in Ovid MEDLINE to “Complete Reference” makes the Title comment field visible on the aggregate search results page from which a searcher can view the citation to the published article. One may be able to limit to those records that have a link to a published article. A searcher could presumably use the following command using the NOT Boolean operator in preprint-specific search results to remove all preprint records that have a published version: NOT “Update in”.cm.
- Similar to Ovid MEDLINE, EBSCO MEDLINE has the citation of the final journal-published article in the Comment field, preceded by the text “Update in.” The Advanced search screen has a selectable drop-down option for search fields, and the relevant field is called “Comments & References.” Similar to the command provided for Ovid MEDLINE above, a searcher could search for the exact phrase “update in” in the Comments & References field (CR) to potentially limit to or filter out preprint records that have updated citations.
- In PubMed, the link to the journal publication is prominently displayed on the record, both in the aggregate search results and the individual records immediately below the title, author and identifier information, within the section called “Update in.”
- In Preprint Citation Index, the number of versions and the link to the published article (if available) is visible on the aggregate results page, and a notice at the top of the individual record page also indicates the availability and link to the published version. But a method to filter in or filter out these records does not appear to exist.
- In OSF Preprints, the link to the journal-published version, when available, is visible in the full record view only and not in the aggregate search results page view. We had difficulty locating many preprints with links to the published article, which made us wonder whether, in this source, authors were responsible for providing this information, rather than the source server.
- In Google Scholar, manuscript versions are grouped and accessible by selecting “All versions,” but when users use command line searching to isolate preprints any grouping connecting a preprint to an affiliated journal version of record is filtered out, likely because the primary source for that title is the journal-published version
- In bioRxiv/medRxiv, the information for the journal-published article is visible on the individual record page but not on the search results page. According to their FAQs, the server attempts to locate the published copy.^34^

### 4.3 Reporting bias

While Europe PMC, MEDLINE (EBSCO), and MEDLINE (Ovid) include functionality to enable limiting to preprints not affiliated with a journal article, this approach may not be appropriate for reasons such as reporting bias, and selective or suppressive dissemination of research. The PRISMA 2020 Statement instructs authors to report the total number of reports and the total number of studies.^35^ There are instances where one report contains multiple studies or where the same studies are represented across multiple reports. The preprint and journal-published versions are considered to be distinct reports of the same study. Tools like Covidence can merge and unmerge reports related to the same study for data extraction. Research shows a low rate of change between preprints and journal article versions, but there are scenarios where a preprint reports a variable that is then omitted or reported differently in the peer review version.^36^ ^–^ ^42^ For this reason, teams should include all available iterations of a study and not favor the peer-reviewed version.

## 5 Recommendations for practice

### 5.1 Freely accessible aggregator options

When research teams are under-resourced, freely accessible sources may be the only accessible options. Presently, seven sources from our sample are free to use: medRxiv/bioRxiv, Europe PMC, Google Scholar, OSF Preprints, PubMed, Lens.org, and OpenAlex. Of these options, Europe PMC, PubMed, and Lens.org all contained the desirable qualities with OpenAlex also scoring well. Note that PubMed’s coverage of preprint records is incomplete, and therefore we do not recommend the use of PubMed for comprehensive preprint searching. For this reason, we recommend Europe PMC, Lens.org, and OpenAlex.

### 5.2 Considerations for selecting aggregators to search

1. The choice of aggregator depends on your topic. Searchers should know which preprint servers exist and are most relevant in their disciplines. Searchers could gain familiarity of these from directories or listings of preprint servers or repositories. Three such lists include:
  - ASAPBio’s List of Preprint Servers: Policies and Practices across Platforms (https://asapbio.org/preprint-servers). This directory includes disciplinary scope as well as where the content is indexed.
  - Directory of Open Access Preprint Repositories (DOAPR https://doapr.coar-repositories.org/repositories/) which features disciplinary scope, languages, and content types among other details. Each server is listed in the “Repositories” tab. Note that older entries on DOAPR may not have been updated recently. For example, as of February 27, 2025, the entry for arXiv shows a last update date of June 2, 2021.^43^
  - Wikipedia also has a listing of preprint servers
    (https://en.wikipedia.org/wiki/List_of_preprint_repositories).
  The next two considerations, selecting an aggregator based on coverage and selecting the aggregator based on minimum search functions required for the search strategy, do not necessarily need to be done in the order presented below.
2. When multiple aggregator options exist that provide the server coverage relevant to a topic or discipline, the decision will depend on the search functions that the search requires. Some searches may have a relatively simple search strategy that requires few advanced features (such as proximity, wildcards, and multiple levels of nesting parenthesis) or have a small enough set of terms such that truncation is not a critical operator, and in those cases, there would be a greater number of aggregators to choose from. However, searches for more complex topics may require advanced search functions to construct a sufficiently sensitive search strategy, and this may reduce the options available to the searcher. A searcher can use the results of Tables 4 and 5 in this article to aid in decision-making.
3. Since aggregators may offer superior search functionality, the next step would be to identify the aggregator options that include coverage of your desired list of preprint servers. The Directory of Open Access Preprint Repositories has a list of “Integrated services,” including search indexing where the number and names of preprint servers included in a source can be found. Similarly, they have a listing of each repository and where it is indexed. Unfortunately, many of the pages in this resource have not been updated since 2021. For example, the DOAPR page on Europe PMC states that 15 servers are included, whereas Europe PMC’s website states it includes records from 34 servers (with partial coverage from 2 servers).^44^ If this resource were kept up to date, it would serve as an authoritative resource and starting point for determining which sources or aggregators are available for consideration by the searcher.

### 5.3 Preprint source decision scenarios

As searchers contemplate searching for preprints, we present two example scenarios below to demonstrate the decision-making process (Table 7).

**Table 7:** Two search scenarios to assist in preprint source decision-making Table 7 long description.

**Scenario 1**	
Context:	A health librarian working with a team on a medical systematic review who is primarily interested in searching medRxiv.
Aggregators with relevant coverage:	Based on the coverage tests presented in Table 6, Europe PMC, OpenAlex, Scopus, and Web of Science PCI have reasonable coverage of medRxiv.
Considerations based on features:	Europe PMC and Web of Science have all essential features. Scopus is missing the key essential feature of batch exporting which renders it unusable. OpenAlex has 12 essential features, but the web interface lacks command line searching. The best option, which is freely accessible, includes all essential features, and has the highest coverage of medRxiv, is Europe PMC. However, it lacks the proximity function. If the topic of the review has complex and diverse terminology and thus the ability to use proximity is critical for this search, then Web of Science PCI may be the best option. While OpenAlex has a proximity operator, its inability to combine truncation with the proximity operator and the character limit of 2,048 may impact its usability for a complex review.
**Scenario 2**	
Context:	A social sciences (organizational psychology) librarian working with a team on an interdisciplinary systematic review is primarily interested in including a few relevant preprint servers including PsyArXiv, RePec, OSF Preprints.
Aggregators with relevant coverage:	After reviewing Table 1 and aggregator help pages, the librarian determines these aggregators have some of the desired servers: Europe PMC (PsyArXiv) OpenAlex (RePeC) Lens.org (PsyArXiv, OSF Preprints) No single source has all 3 of the desired servers, so a combination of Lens.org and OpenAlex would be the minimum combination required. Of these two, OSF Preprints is only covered in Lens.org, which also provides coverage of PsyArXiv (since it is a community repository within the OSF Preprints platform). RePeC is only available in OpenAlex (among the aggregators we studied).
Considerations based on features:	Lens.org has all 13 of the essential features and has a limited proximity function (which is a key desirable feature for complex searches). OpenAlex has 12 of the 13 essential features, also with a limited proximity feature but no command line functionality in the web interface. If both aggregators are to be searched, it may be more appropriate to search PsyArXiv and OSF Preprints in Lens.org, then RePeC preprint records in OpenAlex, to get the optimal combination of coverage and functionality. Since Europe PMC provides no additional features compared to Lens.org, there is no case to be made for searching for PsyArXiv records using Europe PMC, which would only add search translation burden to the searcher. Note that we did not include OSF Preprints (the aggregator) in the initial list for consideration because it lacks any export functionality.

### 5.4 How to limit to preprints (as a type) and to specific preprint servers

Sources permit either limiting to preprints as a publication type or limiting to specific preprint servers. How the user executes this search varies greatly by platform and often is not intuitive. Table 8 shows steps for limiting at the publication type or server-level.

**Table 8:** Commands or instructions on how to limit to preprints as well as specific servers for each source Table 8 long description.

Source	Command to limit results to preprints	Command to limit to by server(s)
Europe PMC	AND (SRC:PPR)Or,Use the Preprints checkbox in left-hand filters.	AND ((PUBLISHER:“servername”)Or,Sometimes, AND (JOURNAL:“servername”)) Note: When applying this limit, check if ((PUBLISHER:“servername”) OR (JOURNAL:“servername”)) finds more results than Publisher alone; and check if removing AND SRC:PPR finds more preprints.
Embase (Ovid)	AND preprint.pt Or,Limit <line number> to preprint	AND servername.jn Or,Filter by journal title on left.
Medline (Ovid)	AND preprint.pt Or,Limit <line number> to preprint	AND servername.jn Or,Filter by journal title on left.
Medline (EBSCO)	Advanced search: search “preprint” in the Publication Type field (selectable from drop-down).	Filter by publication on left.
PubMed	AND preprint[pt]Or,Advanced search: preprint in Publication Type field.	AND “servername”[Journal]
Preprint Citation Index	Navigate to: “Preprint Citation Index” and then search normally	AND SO = servername Or,Filter by Repositories on the menu on the left.
Scopus	From the search results, click on the tab Preprints (beta)	Filter by Repository on left.
OSF Preprints	Go directly to: https://osf.io/preprints (only preprints)Or, from the OSF main page, select the Preprints tab from the menu at the top left.	Filter by Provider on left.
Lens.org	In filters, select Document type = preprint Or, in the Query text box, type publication_type:preprint	Filter by Journal (or in some cases Publisher) on left Or,Search the Source Title field (or the Source Publisher field) in the query editor.Notes:Use truncation, e.g., medRxiv*, and check for spelling variations.If you need to limit to preprint servers that are listed in two different facets, then use the text query editor. For example, the following can be used to include both Authorea and arXiv: ((source.publisher: “authorea”) OR (source.title: “arxiv”))
Google Scholar	NA	site:serverurl (e.g., site:medRxiv.org)Or,source:servername (e.g., source:medRxiv)
bioRxiv/medRxiv	NA (only preprints)	You can go directly to medrxiv.org or biorxiv.org to search either separately, or select either or both from the Advanced search.Note: Clear the box first before picking “bioRxiv and medRxiv” in Advanced search. Having one of the databases repeated tends to give unexpected results.
OpenAlex	The main OpenAlex homepage search box is less intuitive to search. Clicking “Enter” takes you to a more sophisticated interface.If you are in the “Advanced” filter style screen:Click “+ Add filter” and select Type. Then check the box next to Preprint Or, if you are in the “Basic” filter style screen:Click the arrow next to “Type” and check the box next to Preprint from the resulting list.You can use the Type filter to limit to preprints before or after you execute a Boolean search query.	In you are using the “Advanced” filter style:Click on the plus (+ Add filter) to add a filter, then scroll (or click more) until you locate “Source,” and select it. The source window will then load which lists the sources which can be selected using checkboxes. You can also use the search box at the top of the window to locate the source.Note: previously, there was a filter category called Repository, which (as of Feb. 4, 2025) is no longer visible

### 5.5 Limitations and directions for future research

This research is a snapshot in time because aggregators can change with or without warning. The landscape is also evolving with new preprint servers and discovery options coming to fruition, while some preprint servers have ceased operation.

We did not test the quality or structure of exported results. This can potentially impact the ability to deduplicate results across aggregators. Future research should explore the quality of indexing, metadata of exports, and implications for deduplication in detail.

The coverage tests in this study were exploratory and focused on the volume of medRxiv records available in each aggregator. However, additional research is needed to understand coverage more fully including how the sources and aggregators respond to versioning. Future confirmatory research could explore comprehensiveness of coverage, disciplinary focus, and recall using investigative methods.

## 6 Conclusions

We investigated the search functionality and explored coverage of 12 preprint aggregator sources. We found substantial variability across platforms in regard to search functionality which has implications for evidence synthesis searchers. Based on the findings and challenges that we encountered during our study, we have presented comparison tables, recommendations, and scenarios to aid decision-making for searchers. More research is needed on aspects of information retrieval of preprint records in the context of evidence synthesis work.

## Data Availability

The materials and detailed data collected are available at the following repository: https://doi.org/10.5683/SP3/VSML4B.

## Long descriptions

### Table 1 Long description

The table has four columns: Database name, Number of preprint servers included, Free or subscription, and URL if free access. From top to bottom: medRxiv and bioRxiv combined includes 2 servers, is free, URL is https://www.medrxiv.org/search. Embase (Ovid and Embase.com) includes 3 servers, subscription required, no URL. Europe P M C includes 34 servers, is free, URL is https://europepmc.org. Google Scholar includes many servers, is free, URL is https://scholar.google.com/. O S F Preprints includes 29 servers, is free, URL is https://osf.io/preprints/. PubMed and M E D L I N E row is subdivided: PubMed includes preprints with N I H affiliation from 4 servers, is free, URL is http://pubmed.gov; M E D L I N E (E B S C O) and M E D L I N E (Ovid) are subscription only, no URLs. Scopus includes 7 servers from 2017 onward, subscription required, no URL. Web of Science Preprint Citation Index includes 5 servers, subscription required, no URL. Lens dot org does not provide a server list, is free for up to 1,000 records, URL is https://www.lens.org/. OpenAlex does not provide a server list, is freemium, URL is https://openalex.org/.

Navigate back to Table 1.

### Table 2 Long description

The header row contains five columns labeled ID, Category, Search function, Points, and Description. The table has thirteen main rows plus two sub-rows for item 8. Row 1, ID 1, Category Database feature, Search function Total results displayed, Points 1, Description The exact total number of results is displayed. Row 2, ID 2, Category Database feature, Search function Bulk download/export, Points 1, Description Ability to download all results in batches. Row 3, ID 3, Category Query development, Search function Command line searches, Points 1, Description Ability to write searches using Boolean syntax and field codes. Row 4, ID 4, Category Query development, Search function High character or word limits, Points 1, Description Character or word limit for the query box. Row 5, ID 5, Category Query development, Search function Boolean operators, Points 1, Description Supports AND, OR, NOT operators. Row 6, ID 6, Category Query development, Search function Nesting, Points 1, Description Parentheses can set Boolean operation order. Row 7, ID 7, Category Query development, Search function Field searching, Points 1, Description Provides field codes or drop-down list for data fields. Row 8a, ID 8a, Category Query development, Search function Title field search, Points 0.5, Description Search terms limited to title field. Row 8b, ID 8b, Category Query development, Search function Abstract field search, Points 0.5, Description Search terms limited to abstract field; if no separate abstract field, title or abstract search receives full score. Row 9, ID 9, Category Query development, Search function Truncation (right-hand truncation), Points 1, Description Ability to truncate search term with a symbol for variant endings. Row 10, ID 10, Category Query development, Search function Exact phrase searching, Points 1, Description Ability to search for exact phrase with same word order. Row 11, ID 11, Category Query development, Search function Exact single term searching, Points 1, Description Ability to turn off lemmatization or stemming. Row 12, ID 12, Category Query filters and limits, Search function Can you limit to preprints, Points 1, Description Ability to limit search to preprints. Row 13, ID 13, Category Additional features, Search function Reproducibility at different locations, Points 1, Description Same search can be executed by different people in different locations and obtain same results. Each row is color-coded by category: Database feature (blond), Query development (carolinablue), Query filters and limits (babypink), Additional features (cambridgeblue).

Navigate back to Table 2.

### Table 3 Long description

The header row contains four columns labeled ID, Category, Search function, and Description. Starting from the top row, ID 1 is under Query development with Multi-line search, described as combining multiple search lines from history for complex Boolean searches. ID 2, Query development, Wildcards, allows retrieval of term variations using optional or mandatory wildcard symbols, such as an?emia for anemia or anaemia. ID 3, Query development, Hyphens, tests if hyphens and spaces are treated interchangeably in search terms, e.g., socio-economic versus socio economic. ID 4, Query development, Proximity, enables searching for two terms within a specified number of words, with operators placed either between terms or at the end of phrases. ID 5, Query development, Truncation within a proximity string, combines right-hand truncation with proximity searching. ID 6, Query development, Controlled vocabulary (subject headings), tags preprints with controlled vocabulary and allows Boolean combination with free text searching. ID 7, Query filters and limits, Limit to specific servers, restricts searches to particular preprint servers. ID 8, Query filters and limits, Can you exclude published preprints, asks if published preprints can be excluded from results. ID 9, Query filters and limits, Date limits, restricts searches to specific date or year ranges. ID 10, Additional features, Link to the final (journal) publication, provides a direct link to the final publication without navigating through the preprint server.

Navigate back to Table 3.

### Table 4 Long description

The table has 13 rows for aggregators and 16 columns for features. From left to right, columns are Source, Number of results displayed, Bulk export, Command line search, High word or character limit, Boolean operators, Parentheses or nesting, Field search, Title search, Abstract search, Truncation, Exact phrase search, Exact single term, Limit to preprints, Reproducibility, and Number of essential features out of 13. For each aggregator, cells are marked Yes, No, Other, or Yes with an asterisk, indicating feature presence. Embase (Ovid), Europe P M C, Lens dot org, MEDLINE (EBSCO), MEDLINE (Ovid), Preprint Citation Index, and PubMed have Yes in all 13 feature columns. bioRxiv and medRxiv have 8 Yes, Google Scholar 6.5, OpenAlex 12, OSF Preprints 9, and Scopus 12. ‘Other’ in Abstract search means the field is only available combined with Title. Asterisk in Reproducibility means the feature appears to work but is unconfirmed. Caret in OpenAlex means the feature is API-only. The table footnotes explain these notations.

Navigate back to Table 4.

### Table 5 Long description

The table has thirteen rows for sources: bioRxiv and medRxiv, Embase (Ovid), Europe P M C, Google Scholar, Lens dot org, MEDLINE (E B S C O), MEDLINE (Ovid), OpenAlex, O S F Preprints, Preprint Citation Index, PubMed, and Scopus. Columns, from left to right, are: Multi-line searching, Wildcards, Hyphen equals space, Proximity searching, Truncation with proximity, Controlled vocabulary assigned, Limit to specific servers, Exclude published preprints, Ability to limit by date, and Links to published version. For each aggregator, cells are marked Yes, No, N A, or Other. Embase (Ovid), M E D L I N E (Ovid), and Scopus have Yes for most features except Controlled vocabulary assigned and Exclude published preprints. bioRxiv and medRxiv lack Multi-line searching, Proximity searching, and Exclude published preprints, but support Wildcards, Hyphen equals space, Controlled vocabulary assigned, Limit to specific servers, Ability to limit by date, and Links to published version. Europe P M C and Google Scholar lack most features except Hyphen equals space, Limit to specific servers, Ability to limit by date, and Links to published version. Lens dot org supports most features except Truncation with proximity, Exclude published preprints, and Links to published version. OpenAlex and O S F Preprints lack Multi-line searching, Proximity searching, Truncation with proximity, Controlled vocabulary assigned, and Exclude published preprints. Preprint Citation Index supports all except Controlled vocabulary assigned and Exclude published preprints. PubMed supports most features except Wildcards, Truncation with proximity, Controlled vocabulary assigned, and Exclude published preprints. Table footnotes clarify that ‘Other’ in Exclude published preprints means the feature is not explicit but may be approximated, and for Google Scholar, preprints are excluded by default when searching for preprint sources.

Navigate back to Table 5.

### Table 6 Long description

From the top row downward, the table lists sources in descending order of total preprint coverage as of October 31, 2024. Columns are Source, Total preprint coverage, Total medRxiv coverage, Total medRxiv coverage as a percent of the medRxiv coverage, 2022 medRxiv coverage, and 2022 medRxiv coverage as a percent of the 2022 medRxiv coverage. OpenAlex leads with 6,398,000 total preprints, 56,580 medRxiv, 94.44 percent, 9,988 for 2022, and 92.40 percent. Lens dot org follows with 3,909,816 total, 5,581 medRxiv, 9.32 percent, 334 for 2022, and 3.09 percent. Scopus has 2,462,629 total, 56,785 medRxiv, 94.78 percent, 10,426 for 2022, and 96.45 percent. Preprint Citation Index shows 2,438,003 total, 38,152 medRxiv, 63.68 percent, 7,748 for 2022, and 71.67 percent. Europe P M C has 858,887 total, 59,684 medRxiv, 99.62 percent, 10,517 for 2022, and 97.29 percent. bioRxiv and medRxiv, highlighted in green as the gold standard, report 315,102 total, 59,910 medRxiv, 100 percent, 10,810 for 2022, and 100 percent. Embase Ovid lists 143,107 total, 32,098 medRxiv, 53.58 percent, 10,430 for 2022, and 96.48 percent. MEDLINE Ovid, PubMed, and MEDLINE EBSCO each have about 31,539 total, 5,638 medRxiv, 9.41 percent, 335 for 2022, and 3.10 percent. Google Scholar, with approximate values highlighted in red, shows not available for total, about 82,800 medRxiv, not available for percent, about 6,260 for 2022, and not available for percent. OSF Preprints, highlighted in gray, has not available for all columns. Gold standard values are marked with an asterisk and green, approximate values with a tilde and red, and unavailable values with gray. The table footnote explains these highlights.

Navigate back to Table 6.

### Table 7 Long description

The table is divided into two main sections, Scenario 1 and Scenario 2, each spanning two columns. Scenario 1, at the top, addresses a health librarian conducting a medical systematic review focused on medRxiv. The context row states the librarian’s primary interest in medRxiv. The next row, aggregators with relevant coverage, lists Europe P M C, OpenAlex, Scopus, and Web of Science P C I as having reasonable medRxiv coverage. The feature considerations row uses bullet points: Europe P M C and Web of Science have all essential features; Scopus lacks batch exporting, making it unusable; OpenAlex has 12 essential features but lacks command line searching in its web interface. The narrative concludes that Europe P M C is the best freely accessible option with all essential features and highest coverage, but lacks proximity function. If proximity is critical, Web of Science P C I is preferred. OpenAlex’s proximity operator is limited by inability to combine with truncation and has a 2,048 character limit which limits its usefulness in a complex review. Scenario 2, below, describes a social sciences librarian seeking to include PsyArXiv, RePEc, and O S F Preprints. The context row explains the interdisciplinary review. Aggregators with relevant coverage are listed as Europe P M C (PsyArXiv), OpenAlex (RePEc), and Lens dot org (PsyArXiv, O S F Preprints). No single aggregator covers all three servers; a combination of Lens dot org and OpenAlex is required. Lens dot org covers PsyArXiv and O S F Preprints, OpenAlex covers RePEc. Feature considerations, in bullet points, state Lens dot org has all 13 essential features and limited proximity function; OpenAlex has 12 features, limited proximity, and no command line in the web interface. The narrative recommends searching PsyArXiv and O S F Preprints in Lens dot org, RePEc in OpenAlex, and notes Europe P M C adds no additional features for PsyArXiv. O S F Preprints aggregator is excluded due to lack of export functionality.

Navigate back to Table 7.

### Table 8 Long description

The table has three columns. The first column, Source, lists Europe P M C, Embase (Ovid), Medline (Ovid), Medline (E B S C O), PubMed, Preprint Citation Index, Scopus, and O S F Preprints. The second column, Command to limit results to preprints, details for each source: Europe P M C uses ‘A N D (S R C colon P P R)' or the Preprints checkbox in left-hand filters; Embase (Ovid) and Medline (Ovid) use ‘A N D preprint dot p t’ or ‘Limit less than line number greater than to preprint’; Medline (E B S C O) uses advanced search for ‘preprint’ in the Publication Type field; PubMed uses ‘A N D preprint open bracket p t close bracket’ or advanced search for preprint in Publication Type; Preprint Citation Index requires navigating to ‘Preprint Citation Index’ and searching; Scopus uses the Preprints (beta) tab; O S F Preprints directs users to ‘h t t p s colon forward slash forward slash o s f dot i o forward slash preprints’ or the Preprints tab. The third column, Command to limit to by server(s), specifies for each source: Europe P M C uses ‘A N D open parenthesis PUBLISHER colon servername close parenthesis’ or ‘A N D open parenthesis JOURNAL colon servername close parenthesis’, with a note to compare results and check if removing ‘A N D S R C colon P P R’ finds more preprints; Embase (Ovid) and Medline (Ovid) use ‘A N D servername dot j n’ or filter by journal title on the left; Medline (E B S C O) filters by publication on the left; PubMed uses ‘A N D servername open bracket Journal close bracket’; Preprint Citation Index uses ‘A N D S O equals servername’ or filters by Repositories on the left; Scopus filters by Repository on the left; O S F Preprints filters by Provider on the left.

Navigate back to Table 8.
